# Anxiety and depression symptoms, albuminuria and risk of acute myocardial infarction in the Norwegian HUNT cohort study

**DOI:** 10.1186/s12872-022-02921-1

**Published:** 2022-11-08

**Authors:** Lise Tuset Gustad, Tor Åge Myklebust, Ottar Bjerkeset, Lana J. Williams, Lars Erik Laugsand, Håvard Dalen, Michael Berk, Solfrid Romundstad

**Affiliations:** 1 Faculty of Nursing and Health Sciences, Nord University, Levanger, Høgskoleveien 27, 7601 Levanger, Norway; 2 Department of Circulation and Medical Imaging, Medicine and health sciences, Norwegian University of Science and Technology (NTNU), Trondheim, Norway; 3 Department of Internal Medicine, Levanger Hospital, Nord-Trøndelag Hospital Trust, Levanger, Norway; 4 Department of Registration, Cancer Registry of Norway, Oslo, Norway; 5 Department of Research and Innovation, Møre and Romsdal Hospital Trust, Ålesund, Norway; 6 Department of Mental Health, Faculty of Health and Medicine, Norwegian University of Science and Technology (NTNU), Trondheim, Norway; 7 Deakin University, IMPACT – the Institute for Mental and Physical Health and Clinical Translation, School of Medicine, Barwon Health, Geelong, Australia; 8 Department of Emergency Medicine, St. Olavs Hospital, Trondheim University Hospital, Trondheim, Norway; 9 Clinic of Cardiology, St. Olavs Hospital, Trondheim University Hospital, Trondheim, Norway; 10 Orygen, The National Centre of Excellence in Youth Mental Health, Centre for Youth Mental Health, Florey Institute for Neuroscience and Mental Health and the Department of Psychiatry, The University of Melbourne, Melbourne, Australia; 11 Department of Clinical and Molecular Medicine, Norwegian University of Science and Technology, Trondheim, Norway

**Keywords:** Depression, Albuminuria, Risk, Cardiovascular disease, Mood disorder, Anxiety, Mental disorders, Neuroscience, Comorbidity, Psychiatry, Renal disease

## Abstract

**Background:**

Studies suggest increased risk for an outcome in people with joint exposures that share common causal pathways. The objective of this study was to determine the risk of incident acute myocardial infarction (AMI) following exposure to both albuminuria and/or anxiety and depression symptoms.

**Methods:**

Participants who provided urine samples to the HUNT2 (1995–97) or HUNT3 (2007–2009) surveys were followed until the end of 2016. Albuminuria was measured by Albumin Creatine Ratio (ACR) and participants self-reported mood and anxiety symptoms on the Hospital Anxiety and Depression scale. We used Cox regression to estimate hazard ratios (HRs) for first incident AMI considering interaction between exposures and additive models to calculate the proportion of AMI that were attributable to the synergy of both exposures, adjusted for the Framingham variables.

**Results:**

Eleven thousand fourteen participants free of previous AMI were eligible for participation, with 1234 incident AMIs occurred during a mean 13.7 years of follow-up. For participants who had a healthier CVD risk profile, the HR for AMI of having both albuminuria (3–30 mg/mmol) and depression (≥8) was 2.62 (95% 1.12–6.05) compared with a HR 1.34 (95% CI 1.04–1.74) with raised ACR only (Likelihood Ratio-test 0.03). Adding anxiety (≥8) to albuminuria (3–30) tripled the risk (HR 3.32 95% CI 1.43–7.17). The additive models suggest that these risks are not higher than expected based on each risk factor alone.

**Conclusions:**

This study indicate that the risk of AMI in persons with elevated albuminuria but with an otherwise healthy CVD profile might be amplified by anxiety and depression symptoms. The increased risk with joint risk factors is not higher than expected based on each risk factor alone, which indicate that the risk factors do not share causal pathways.

**Supplementary Information:**

The online version contains supplementary material available at 10.1186/s12872-022-02921-1.

## Key messages

- Additional depression or anxiety symptoms to albuminuria gives respectively 2- and 3-fold heightened risk of future AMI in younger persons with a healthy cardiovascular risk profile.
- Our findings indicate that the progression of neuropsychiatric disorders, neuroprogression overlap with progression of somatic disorders, somatoprogression.
- Future studies should assess if interventions targeted to the group at risk would decrease AMI risk.

## Background

Despite recent medical advances and a decline in overall incidence, acute myocardial infarction (AMI) remains a major public health problem [1]. The traditional risk factors for cardiovascular disease described in the Framingham Heart Study are age, sex, diabetes, antihypertensive treatment, systolic blood pressure, smoking and lipids (total cholesterol and HDL cholesterol) [2]. However, both chronic kidney disease and severe mental illness have been identified as independent risk factors for AMI in the updated QRISK3 [3], another cardiovascular disease risk prediction tool. For kidney disease, an increased risk of AMI is observed from the early asymptomatic stages detected by an elevated Albumin Creatine Ratio (ACR) in urine (albuminuria) [4]. Even low levels of albuminuria (previously called microalbuminuria) [5–8] are associated with increased risk of AMI, and this risk increases linearly with increasing ACR [9, 10]. For mental illness, self-reported symptoms of depression are associated with a mild to moderate increased risk for AMI [11] and a formal diagnosis is likely to double the risk [12, 13]. This gradient in risk of AMI from symptoms to diagnosis of depression is thought to represent a dose-response effect of the exposure [11] and mental distress such as anxiety and depression show risk gradients comparable to that of elevated cholesterol [14]. Whilst the evidence regarding the connection between depression and AMI is well established [15], a more conflicting evidence base exists regarding anxiety symptoms [16]. However, depression and anxiety, both at symptomatic and diagnostic levels, often coexist, and this coexistence often represents a more severe phenotype than single condition alone [13, 16].

In circumstances with shared biological pathways towards disease, the risk of having both exposures would be expected to be higher than the addition of the separate effect of each exposure [17–19]. There is evidence for shared pathophysiological pathways of depression/anxiety, albuminuria and cardiovascular diseases, where inflammation and endothelial dysfunction are central for several conditions [16, 20, 21]. However, today the risk for disease is mostly assessed with single conditions and not based on synergistic effects [17].

Therefore, we aimed to evaluate and compare the risk of AMI in people with single or joint exposures of elevated ACR and depression or anxiety symptoms in persons free of CVD or overt kidney disease. This hypothesis has not yet been evaluated and could be of clinical importance to reduce the increased risk of AMI for persons with depression or anxiety symptoms or with elevated ACR levels or both.

## Methods

### Study population

The HUNT Study regularly invites the total adult population in the northern county of Trøndelag (≥20 years) for an extensive health screening [22]. Data collected includes information on sex, age, clinical examinations, blood samples and self-reported health-related questionnaires. In HUNT2 (1995–1997), 93,898 persons were invited with 65,004 (69.2%) agreeing to participate and in HUNT3 (2006–2008), 93,860 were invited with 50,663 (53.9%) agreeing to participate.

### The albuminuria sub-study

HUNT2 and HUNT3 participants were invited to a sub study where morning urine samples were collected for ACR analysis. Participants were selected according to predefined selection criteria: i) a 5% random selection of the HUNT participants, ii) those with self-reported diabetes (yes/no), iii) those with self-reported hypertension and/or self-reported antihypertensive treatment (yes/no) (HUNT2 only), and; iv) in HUNT3, the HUNT2 albuminuria study participants were re-invited [23, 24]. In this paper, participants from selection group iv) were only included once (HUNT2 participation date). The study participants were asked to provide urine samples over three consecutive days and answered questionnaires related to the urinary samples including history of a urinary tract infection during the last week, persistent haematuria over the last year, and whether women were pregnant or menstruating at collection time [23]. Of those invited to the albuminuria study 84.1 and 63.3% participated in HUNT 2 and HUNT3, respectively.

### Clinical examination

Trained nurses undertook standardised clinical examinations at baseline. The examinations included measurements of the participants height, waist circumference and weight (all without shoes and wearing light clothing, to the nearest cm or kg) [22]. Body mass index was calculated as kg/m^2^. We used the average of second and third systolic blood pressure (mmHg) and pulse (heart rate/minute) measurements, recorded by an automatic oscillometric method (Dinamap 845XT; Criticon, Tampa, Florida, USA) after > 5 minutes resting in a sitting position [22].

### Laboratory data

Blood sampling was performed in a non-fasting state at the time of clinical examination. Serum total and high-density lipoprotein (HDL) cholesterol were analysed using enzymatic colorimetric methods (Boeheringer Mannheim, Germany) [22]. Urine samples were returned by the HUNT participants using prepaid envelopes and standardised receptacles. Fresh blood and urine samples were analysed at an accredited laboratory (ISO-9001 certified and ISO/IEC-17025) at Levanger Hospital (Norway). For the analysis, HUNT2 used a Hitachi 911 autoanalyzer (Hitachi, Mito, Japan) with reagents from Boehringer Mannheim (Mannheim, Germany) and HUNT3 used an Architect ci8200 autoanalyzer (Abbot Diagnostic, Longford, Ireland) with reagents from Mulitigent (Abbot Laboratories, USA) [22].

HUNT used the Jaffe ´ method to measure serum concentration of creatinine, calibrated to isotope-dilution mass-spectroscopy. Creatinine was thereafter used to calculate the estimated glomerulus filtration rate (GFR, ml/min), using the Chronic Kidney Disease Epidemiology Collaboration equation (CKD-EPI) .

Immunoturbidimetric methods were applied to determine urine albumin using antihuman serum albumin, and ACR was calculated in mg/mmol. In HUNT2, the supplier was DakoAS, Glostrup, Denmark [25] and in HUNT3, Abbot Laboratories [22]. All information on quality control and calibration methods is previously published [23].

### Self-report measures

Participants self-reported anxiety and depression symptoms experienced over the past week using the Hospital Anxiety and Depression rating scale (HADS). The HADS includes 14 questions, 7 mirroring non-somatic depression symptoms from the ICD depression criteria [26], such as anhedonia and psychomotor retardation, and 7 anxiety questions with one item mirroring panic disorder and the other six general anxiety – including worrying and rumination [27]. The HADS is licenced [27] and HUNT obtained licences in order to use in each survey. Each question is answered according to symptom severity using a scale from 0 (no symptoms) to 3 (high symptom load), i.e. each subscale ranges 0–21 points.

Use of antihypertensive medication and diabetes was self-reported (yes/no). Smoking status was reported as never, previously or current. Self-reported diabetes was categorised as yes or no, and antihypertensive medications use was categorised as yes (current or previous) or no (never) [22]. Physical activity was self-reported as hours of intensive physical activity per week, defined as physical activity involving sweating or feelings of breathlessness. Physical activity duration was categorised as 0 hours (none), < 1 hour, 1–3 hours and > 3 hours.

We constructed three categories for “education and work” based on self-reported education for HUNT2 participants and Classification of Occupation from Statistics Norway for HUNT3 participants; < 10 years of school or unskilled worker, 10–12 years of school or intermediate worker, > 12 years of education or belonging to the salariat class [22].

### Follow-up and outcome AMI ascertainment

**Fig. 1:**
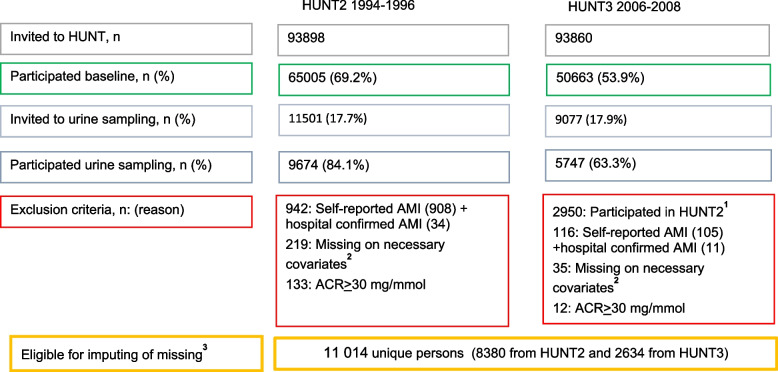
“Flow chart of inclusion of participants to the study”. Abbreviations; HUNT = Nord-Trøndelag Health Study; HUNT2 = Second Wave of HUNT; HUNT3 = Third wave of HUNT. ^1^ Participants in HUNT3 who were reinvited after HUNT2. ^2^ Imputation models did not converge before we excluded those with missing on blood pressure, diabetes, serum cholesterol, bmi, waist circumference, heart rate and Chronic Kidney Disease Epidemiology Collaboration equation (CKD-EPI). ^3^ Persons that participated in urine sampling and did not have any exclusion criterion on other variables than those described in footnote ^2^ is described in Table 1

Of the 15,421 participants who delivered at least one urinary sample in either HUNT2 or HUNT3, we excluded 1058 (6.9%) with previous AMI, 145 (0.9%) with an ACR indicating overt kidney disease (ACR > 30 mg/mmol), 245 (1.6%) with missing data on necessary covariates and participants who did not self-report AMI, but had hospital records indicating an AMI at a date prior to baseline (*n* = 34 (0.2%) in HUNT2 and *n* = 11 (0.1%) in HUNT3). Figure 1 shows the flow of participants in this study. A total of 2950 (19.1%) participants had observations from both HUNT2 and HUNT3 and these were followed-up from their first assessment in HUNT2 (Fig. 1). HUNT3 participants were followed from HUNT3 participation date. Together 11,014 participants were followed-up from their HUNT participation to either a first AMI or untill the end of the follow-up period (31 December 2016). AMI was diagnosed according to the European Society of Cardiology/ American College of Cardiology consensus guidelines [28, 29]. Criteria for AMI included: (i) specific clinical symptoms according to case history information, (ii) changes in blood levels of cardiac enzymes, and (iii) specified ECG changes. The national cause of death registry was another data source, providing the ICD codes for AMI that never reached hospitals using code 410 in the 9th revision and codes I21 and I22 in the10^th^ revision in order to confirm AMI [11].

### Statistical analysis

Participants who provided only one or two urine samples, answered less than 5 items on each HADS subscale, self-reported pregnancy, urinary tract infection, haematuria or menstruation, or had urinary samples with deviations in ACR measurements were set to missing and thereafter imputed. The ACR values were dichotomised as ACR (no/yes) and cut off for normal values (ACR, no) was set at 3 mg/mmol.

Per protocol, we replaced 1–2 missing items on the HADS depression and HADS anxiety subscale with 6/7 and 5/7 of the values provided [11] and defined these as our complete case numbers (see statistical section for more details regarding managing missing data). Dichotomisation of depression symptoms (no/yes) and anxiety symptoms (no/yes) were according to score < 8 (no) or 8–21 (yes) on respective HADS sub-scales as in a previous HUNT study related to additive risk factors [30]. Due to the coexistence of anxiety or depression, a variable “anxiety or depression symptoms” (no/yes) was created, where “yes” was defined as 8–21 in either of the HADS sub-scales [30].

In survival analyses, individuals were followed from baseline measurements (participation in HUNT2 or HUNT3) to date of AMI, death or end of follow-up (31 December 2016), whichever came first. Attained age was used as the underlying timescale. We used multivariable Cox regression models to assess the relative risk and associated 95% confidence interval (95% CI) for incident AMI against three exposures: i) ACR and depression symptoms (*n* = 5599 for Complete Case (CC)); ii) ACR and anxiety symptoms (*n* = 5607 for CC) and; iii) ACR and anxiety or depression symptoms (*n* = 5607 for CC). All exposures (i-iii) were adjusted for confounders between exposures and endpoint in four different models, successively adding confounders to investigate how the HRs of interest changed. We based our statistical models on modern methodology where a-priori knowledge regarding confounders of the association between the exposures and outcome guided the selection and the models were built stepwise to tease out the mechanisms between the exposure and outcome [31]. In Model 1, we adjusted for sex by stratification as this variable otherwise violated proportional hazard assumption (*p* < 0.001). Model 2 included in addition to age and sex the other cardiovascular risk factors from the Framingham Heart Study; diabetes, antihypertensive medication, systolic blood pressure, smoking, total cholesterol and HDL cholesterol. Model 3 further included estimated GFR related to the study selection criteria and body mass index, waist circumference, intensive physical activity and education and work status, which can all be viewed as lifestyle-related confounders. Model 4 included a statistical interaction-term between the exposures of interest; i) ACR and depression; ii) ACR and anxiety; iii) ACR and anxiety or depression. A Likelihood ratio (LR) test to determine interaction were used between model 3 and 4. As the results in model 4 were substantially different in the complete case dataset compared to multiple imputation dataset we ran missing and imputation diagnostics to explore the differences further (Supplementary Table 2). Further, we ran multicollinearity diagnostics using the estat vce, corr option in Stata.

To explore additivity (absolute risk), we defined four mutually exclusive exposure categories based on presence of none, one (either ACR or one of the HADS measures) and both exposure categories. The unexposed to both the first and the second risk factor were defined as reference category. Thus, by defining three indicator variables, three absolute risk coefficients were estimated from a Cox regression model, and the corresponding covariance matrix were used for calculation of confidence intervals. These were plotted in the excel sheet developed by Anderson [32] to calculate the Relative Excess Risk due to Interaction (RERI), Attributable Proportion (AP) of events due to interaction and Synergy Index (S). The latter measure is interpreted as the excess risk from both exposures when interaction is present relative to the risk from exposure when interaction is absent [17]. RERI and AP are statistically significant if the 95% CI do not cross 0, and S is significant if it does not cross 1. Multiple imputation by fully conditional specification were used when imputing missing data, generating a total of 10 complete datasets. Both the “mi chained”-routine and the user-written command smcfcs [33] were used for this purpose.

We used Stata version16.1 for all analysis (StataCorp. 2019. *Stata Statistical Software: Release 16*. College Station, TX: StataCorp LLC).

## Results

Baseline characteristics and proportion of missing data are displayed in Table 1. Of the 11,014 participants, 1234 (11.2%) had a first AMI during the mean 13.7 years of follow-up, or 151,319 person years.

**Table 1:** Baseline characteristics for participants (*n* = 11,014) and overview of missing data

Variables with complete data ***n*** = 11, 014		Missing n (%)
^a^Women (n, %)	6234 (56.6)	NA
^a^Blood pressure medication, never (n, %)	5204 (47.3)	NA
^a^Diabetes, yes (n,%)	1553 (14.1)	NA
^a^Age, years (mean, SD)	57.6 (15.7)	NA
^a^Systolic blood pressure, mmHg (mean, SD)	146.6 (23.8)	NA
^a^Total Cholesterol, mmol/L (mean, SD)	6.2 (1.3)	NA
^a^HDL cholesterol, mmol/L (mean, SD)	1.3 (0.4)	NA
^a^Antihypertensive treatment (yes (n,%)	5810 (52.6)	NA
Estimated glomerulus filtration rate, ml/min (mean, SD)	89.3 (19.5)	NA
Body mass index, kg /m^2^ (mean, SD)	27.9 (4.6)	NA
Waist circumference, cm (mean, SD)	90.8 (12.3)	NA
Pulse, beats/minute (mean, SD)	72.8 (13.4)	NA
**Variables with incomplete data**		**Missing n (%)**
Albumine Creatinine Ratio (ACR), mg/mmol (mean, SD)	2.7 (SD 9.8)	1004 (9.1)
ACR, no (ACR < 3 mg/mmol) (n, %)	8773 (79.7)	
ACR, yes (ACR 3–30 mg/mmol) (n, %)	1237 (11.2)	
HADS Depression Scale (HADS-D, range 0–21) (mean, SD)	3.8 (3.1)	506 (4.6)
Depression Symptoms, no (HADS-D < 8) (n, %)	9159 (83.2)	
Depression Symptoms, yes (HADS-D 8–21 (n, %)	1349 (14.6)	
HADS Anxiety Subscale (HADS-A, range 0–21) (mean, SD)	4.2 (3.4)	741 (6.7)
Anxiety Symptoms, no (HADS-A < 8) (n, %)	8673 (84.4)	
Anxiety Symptoms, yes (HADS-A 8–21) (n, %)	1600 (15.6)	
Anxiety or Depression symptoms, no (n, %)	8360 (78.3)	486 (4.4)
Anxiety or Depression Symptoms, yes (n, %)	2313 (21.7)	
^1^Smoking status (n, %)		226 (2.1)
Never	4973 (46.1)	
Previously	3401 (31.5)	
Current	2414 (22.4)	
Education (HUNT2) or work status (HUNT3) (n, %)		846 (7.7)
10 years of school or less or unskilled worker	4899 (48.2)	
10–12 years of school or intermediate working class	3892 (38.3)	
> 12 years of School or salariat working class	1377 (13.5)	
Intensive exercise (n %)		4355 (39.5)
None	2970 (44.6)	
< 1 hour per week	1518 (22.8)	
1–2 hours per week	1368 (20.6)	
3 hours or more per week	803 (12.0)	

^a^Framingham cardiovascular risk factors

Table 2 shows HR (95% CI) for AMI in models 1 to 3 in the multiplicative cox model after multiple imputation. Models 1 to 3 are without the interaction term between ACR and the HADS measures. In these models an ACR of > 3 was associated with a > 35% increased relative risk for AMI following adjustment for confounders. Without considering additional albuminuria we observed no risk of AMI with symptoms of depression, anxiety or anxiety/depression. All these results were similar in multiple imputation analysis (Table 2) and complete case datasets (Supplementary Table 1).

**Table 2:** Hazard Ratios (HR) and 95% confidence intervals (CI) for AMI during follow-up with exposures

	Model 1	Model 2	Model 3
**i) ACR and HADS-D**			
ACR, no (ACR < 3)	Reference	Reference	Reference
ACR, yes (ACR 3–30)	1.57 (1.35–1.83)	1.40 (1.20–1.63)	1.37 (1.17–1.60)
Depression symptoms, no (HADS-D < 8)	Reference	Reference	Reference
Depression symptoms, yes (HADSD 8–21)	1.12 (0.95–1.31)	1.07 (0.91–1.25)	1.07 (0.91–1.25)
**ii) ACR and HADS-A**			
ACR no (ACR < 3)	Reference	Reference	Reference
ACR yes (ACR 3–30)	1.55 (1.33–1.81)	1.39 (1.18–1.63)	1.35 (1.15–1.62)
Anxiety Symptoms, no (HADS-A < 8)	Reference	Reference	Reference
Anxiety Symptoms, yes (HADS-A 8–21)	1.12 (0.93–1.34)	1.06 (0.91–1.25)	1.07 (0.92–1.26
**iii) ACR and highest score on HADS-D or HADS-A**			
ACR, no (ACR < 3)	Reference	Reference	Reference
ACR, yes (ACR 3–30)	1.58 (1.35–1.84)	1.38 (1.19–1.62)	1.35 (1.16–1.59)
Anxiety or depression symptoms, no	Reference	Reference	Reference
Anxiety or depression symptoms, yes	1.08 (0.94–1.24)	1.01 (0.89–1.20)	1.01 (0.88–1.16)

All results after multiple imputation

Model 1: Age as underlying time scale and Sex as strata

Model 2: Model 2: Model 1 + The Framingham variables diabetes (yes/no), Antihypertensive treatment (yes/no), Systolic Blood Pressure (mmHg), Smoking Status (never, previous, current), total cholesterol (mmol/L), HDL cholesterol (mmol/L)

Model 3: Model2+ Estimated glomerulus filtration rate (mL/min), Body Mass Index (weight/m^2^), Waist Circumference (cm), Intensive Exercise (none, < 1 hr./week, 1–2 hr./week, > 3 hr./week), Education or work (<=10 years school or unskilled worker, 10–12 years school or intermediate working class, > 12 years of school or salariat working class)

Table 3 includes results from the multiple imputation and complete case datasets showing the HRs and 95% CI for future AMI in model 4. Model 4 includes Model 3 plus an interaction term between ACR 3–30 and each of the HADS ≥8 measures in the multiplicative Cox model. Model 4 performed better than model 3 in all analysis (all *p* < 0.01). Results differed between the imputed versus complete cases. In the complete case dataset, having a HADS-D ≥ 8 in addition to an ACR of 3–30 raised the relative risk for future AMI more than two-fold (HR 2.49, 95% CI 1.07–5.70) in comparison to having an ACR of 3–30 alone (HR 1.33, 95% CI 1.02–1.72). In the imputed dataset, the relative HR for AMI with ACR > 3 and additional depression ≥8 was 1.74 (95% CI 0.93–3.25). Similarly, the interaction between having both a HADS-A of ≥8 and an ACR of > 3 raised the relative HR for AMI to 3.41 (95%CI 1.46–7.96) in the complete case dataset. Again, the risk in the imputed dataset was much lower with an relative HR of 1.81 (95% CI 0.99–3.29). The observed differences between the imputed dataset and complete case datasets were related to missing mechanisms on the physical activity questions. Those who had missing on these items had a higher CVD risk compared to those without missing (higher age 62.6 years vs 54.3), higher blood pressure (150.8 vs 143.8), lower kidney function (eGFR 84.6 ml/min vs 92.4), more often diabetes and antihypertensive medication) (Supplementary Table 2). Those who reported on the physical activity question had a strong increased risk of having incident AMI when they had both ACR3–30 and ≥ 8 on any of the HADS measurements (the HR for the interaction between ACR with depression was 1.30, 95% CI 1.10–1.55). This was in strong contrast to those who had missing data on the physical activity questions who had evidence of a reduced risk of future AMI with both exposures (HR for the multiplicative interaction between ACR with depression 0.68, 95% CI 0.29–1.59). The multicollinearity diagnostics showed that the interaction term between exposures had a correlation coefficient of 0.4, all other correlation coefficients were < 0.2.

**Table 3:** Hazard Ratios (HR) and 95% confidence intervals (CI) for AMI with multiplicative interaction between exposures

Imputed models	+HADS-scores < 8	+HADS-Scores ≥ 8
**i) ACR and HADS-Depression**		
ACR yes (ACR > =3)	1.39 (1.04–1.74)	1.74 (0.93–3.25)
**ii) ACR and HADS-Anxiety**		
ACR yes (ACR > =3)	1.36 (1.15–1.60)	1.81 (0.99–3.29)
**iii) ACR and HADS- Depression or Anxiety**		
ACR yes (ACR > =3)	1.33 (1.12–1.59)	1.73 (0.94–3.25)
**Complete Case**		+**HADS-Scores ≥ 8**
**ii) ACR and HADS-Depression**		
ACR yes (ACR > =3)	1.33 (1.02–1.72)	2.49 (1.07–5.70)
**ii) ACR and HADS-Anxiety**		
ACR yes (ACR > =3)	1.30 (1.00–1.47)	3.41 (1.46–7.96)
**iii) ACR and HADS- Depression or Anxiety**		
ACR yes (ACR > =3)	1.23 (0.93–1.62)	2.69 (1.22–5.92)

Model 4: Model 3 (displayed in Table 2) + statistical interaction term (##) between joint exposures of ACR and the different HADS measures i-iii)

Supplementary Table 3 shows the cross-tabulation between exposures of ACR and the HADS measures with number (%) of AMI in the complete case dataset. Whilst 15.5% of the participants with ACR 3–30 and HADS depression < 8 experienced incident AMI, 21.1% of the participants with both elevated ACR and depression scores experienced AMI. When assessing the additive (absolute) risks, we observed no additive risk for AMI with having both albuminuria and either of the HADS measures in complete case nor in imputed datasets (Table 4).

**Table 4:** Evidence for interaction beyond additivity regarding the risk for future Acute Myocardial Infarction (AMI)

	RERI (95% CI)	AP (95% CI)	S (95%CI)
**Imputed models**			
ACR yes (ACR > =3) and Depression yes (HADS-D > =8)	0.36 (−0,34, 1.05)	19.3% (−12.8, 51.4%)	1.73 (0.65, 4.65)
ACR yes (ACR > =3) and Anxiety yes (HADS-A > =8)	−0.37 (− 0,39, − 1.13)	−20.0% (−14.6, −54.7%)	1.77 (0.61, 5.12)
ACR yes (ACR > =3) and HADS-D or HADS-A > =8	0.32 (− 0,62, 1.68)	18.3% (−11.7, 48.4%)	1.78 (0.62, 2,33)
**Complete case**			
i) ACR yes (ACR > =3) and Depression yes (HADS-D > =8)	1.01 (− 0.06, 2.08)	44,3% (14.5, 74.0%)	4.75 (0.84, 26.7)
ii) ACR yes (ACR > =3) and Anxiety yes (HADS-A > =8)	1.48(− 0.98, 1.97)	21.1% (38.4, 68.0%)	5.96 (1.45, 24.5)
iii) ACR yes (ACR > =3) and HADS-D or HADS-A > =8	1.10 (0.73, 1.13)	49,0% (24.1, 73.9%)	8,85 (0.47, 168.0)

Overview of n, events and person-years in CC:

**i)** Albumine Creatinine Ratio (ACR, mg/mmol) and HADS-Depression (*n* = 5660 persons/ 564 cases/ 84,500 person-years)

**ii)** ACR and HADS-Anxiety (*n* = 5599/553 cases/ 83,839 person-years)

**iii)** ACR and HADS- Depression or Anxiety (*n* = 5607/555cases/ 83,951 person-years)

The results on synergisms are derived from model 3 in Table 2

*Abbreviations*: *ACR* Albumine Creatinine Ratio, *RERI* The Relative Excess Risk, *AP* Attributable Proportion, *S* Synergi Index, *CI* 95% Confidence Intervals

## Discussion

In this prospective population based cohort study, the risk of incident AMI in persons with elevated albuminuria and an otherwise healthy CVD profile seems to be amplified by anxiety and depression symptoms. Among younger (mean 54.3 years) participants with fewer cardiovascular risk factors such as diabetes, blood pressure medication and higher glomerulus filtration rate, the multiplicative risk found in the Cox-survival models was two- to three-fold higher when anxiety or depression symptoms (HADS > 8) coexisted with elevated ACR [3–30], compared to those with elevated ACR alone. However, among those of a higher age (mean 62.6 years) with more cardiovascular risk factors, there was no additional risk associated with the presence of anxiety or depression symptoms in addition to elevated ACR. We found no additive risk between the risk factors, suggesting that the observed increased risk from the Cox models is not greater than expected based on each risk factor alone.

Two previous studies suggested that the association between depression and CVD mortality was partially mediated by prevalent incident comorbidity of non-cardiovascular disorder and unhealthy lifestyle behaviours [34, 35]. In our Cox models, we found that this was true only among those of a higher age (mean 62.6 years) who had more cardiovascular risk factors. In studies with data missing not at random, as we observed in model 4, the bias is most likely to occur in the results related to multiple imputation [36], thus we have laid our emphasis on the complete case analysis. The fact that excess risk was evident only among the youngest, more healthy individuals may be explained by the fact that effect of depression on inflammation and endothelial dysfunction might be relatively weak compared to other risk factors such as dyslipidaemia, current smoking, hypertension, diabetes, reduced kidney function [37]. From a public health perspective, this is important given that the risk for AMI was two to three-fold higher among younger persons with few other CVD risk factors when depression or anxiety symptoms were present compared to having an elevated ACR alone. Comorbid cardiometabolic factors and anxiety or depression symptoms may increase the risk of future cardiovascular disease and death [38]. Other studies have found that anxiety and depression symptoms coexist with overall medical burden [39], but when assessing the risk of AMI, albuminuria and mental health measures are often examined separately. We found no study assessing whether anxiety and depression symptoms co-occurring with early signs of kidney disease was associated with increased risk of future AMI. our findings add further evidence to this knowledge base indicating that the progression of neuropsychiatric disorders, neuroprogression overlap with progression of somatic disorders, somatorprogression.

While the Cox models show the relative risks for AMI, the additive models show the absolute risks for AMI with joint exposures [17, 19, 40]. The absence of additive risk for AMI with joint exposures of albuminuria and depression or anxiety symptoms in our study does not indicate a clear support for common causal pathway towards risk for AMI, and that a person with joint risk factors would not have higher risk than expected based on each risk factor alone [14, 15, 28, 41]. Even though our study indicated no evidence of shared causal pathways, evidence from previous studies have shown that the same treatment may ameliorate both albuminuria and mental health measures. Angiotensin-converting enzyme inhibitors and statins which are medications given to reduce AMI risk with elevated ACR, may be promising new targets for treating depression [41–43]. In animal models, the angiotensin-converting enzyme also seems a promising target to alleviate physiologic responses to emotional stress like anxiety [44], a finding reflected in human epidemiological and clinical studies [45–47].

It is a strength to our study that this study explored the joint risk factors and risk of AMI in both a Cox model and an additive model as hypotheses regarding joint risk factors are correctly explored in additive models [17]. A major strength of our study is that the sample includes the whole continuum from young to very old adults. It is shown that endothelial dysfunction is present already from an early age among persons with major depression [48]. Another strength is that the analyses are adjusted for a large numbers of potential cardiometabolic confounders such as blood pressure, diabetes, smoking, body mass index and waist circumference [38].

A limitation to our study is the lower (63% vs 84%) participation rate in HUNT3 vs HUNT2. We cannot exclude some influence on the results, but from a HUNT non responder study it is known that bot very healthy and comorbid individuals were overrepresented among non-responders [49]. Further, the use of self-reported symptoms of anxiety and depression, which is consistently associated with lower effect estimates than formal diagnoses [12, 50]. Thus, a formal diagnosis of anxiety and depression in a young and otherwise healthy individual with elevated ACR could be expected to be associated with an even higher risk of AMI than reported in this study. While the most consistent method for determining excretion of albumin in urine is 24 hours of urine sampling, the use of one or more morning spot urine samples provides good specificity and sensitivity [24]. Another limitation to this study is that the study is observational and cannot prove causality. Anxiety and depression may also affect the risk of AMI in persons with elevated ACR through poorer self-care, lack of treatment adherence or other unmeasured confounders and thereby increase medical burden of albuminuria [39]. As our sample is by selection mostly a multimorbid sample, depression and anxiety symptoms also can co-interact with diabetes and hypertension [51] as we also saw in this study where the risk was higher among participants with a healthier CVD risk profile.

## Conclusions

Our results indicate that younger persons with albuminuria, but without other CVD risk factors, had a 2.49 hazard for future AMI with of additional depression and a 3.41 hazard for future AMI with additional anxiety compared with standalone albuminuria. We did not find any evidence for common causal pathways explaining this interaction. Whether high burden of anxiety and depression symptoms represents a modifiable risk factor for AMI in persons with albuminuria, but an otherwise healthy CVD profile, needs to be tested in future studies.

## Supplementary Information

**Additional file 1: Supplementary Table 1.** Complete Case Analysis related to Table 2. Hazard Ratios (HR) and 95% confidence intervals (CI) for AMI during follow-up. **Supplementary Table 2.** Mechanisms related to missing on the physical activity question. **Supplementary Table 3.** Number (n, %) of participants* without and with Acute Myocardial Infarction (AMI) in Complete Case.

## Data Availability

We do not have ethical approval to deposit our datasets in publicly available repositories. The datasets used and/or analysed during the current study are however available from the corresponding author on reasonable request. All stata codes used will be available from the corresponding author upon reasonable request.

## Abbreviations

ACR: Albumin Creatine ratio
AMI: Acute myocardial infarction
AP: Attributable proportion of events due to interaction
CC: Complete case
CI: Confidence interval
HADS: Hospital anxiety and depression scale
HR: Hazard ratio
HUNT: Nord-Trøndelag health study
MCE: Multiple chained equation
RERI: Relative excess risk due to interaction
S: Synergy index
WC: Waist circumference

## Declarations

### Concent to publication

Consent for publication is given by the data access committee for Nord-Trøndelag Hospital Trust and the HUNT Study on behalf of all participants.
