# Supplementary material for: Anxiety and depression symptoms, albuminuria and risk of acute myocardial infarction in the Norwegian HUNT cohort study
Source: BMC Cardiovasc Disord. 2022 Nov 8;22:472. doi: 10.1186/s12872-022-02921-1 (PMC9644558; doi:10.1186/s12872-022-02921-1)
Supplement: Supplementary file 1 — Additional file 1: Supplementary Table 1. Complete Case Analysis related to Table 2. Hazard Ratios (HR) and 95% confidence intervals (CI) for AMI during follow-up. Supplementary Table 2. Mechanisms related to missing on the physical activity question. Supplementary Table 3. Number (n, %) of participants* without and with Acute Myocardial Infarction (AMI) in Complete Case. [file 12872_2022_2921_MOESM1_ESM.docx]

|  | **Supplementary Table 1.** Complete Case Analysis related to Table 2. Hazard Ratios (HR) and 95% confidence intervals (CI) for AMI during follow-up | | | | |  |
| --- | --- | --- | --- | --- | --- | --- |
|  | | Personyears/ events | Model 1 | Model 2 | Model 3 |  |
| 1. **ACR and HADS-D, n=9544** | |  |  |  |  |  |
| ACR, no (ACR <3), n=8773/ | | 127971/959 | Reference | Reference | Reference |  |
| ACR, yes (ACR 3-30), n=1237 | | 12997/203 | 1.61 (1.37-1.89) | 1.1(1.20-1.67) | 1.46 (1.16-1.85) |  |
| Depression symptoms, no (HADS-D<8) | | 127986/974 | Reference | Reference | Reference |  |
| Depression symptoms, yes (HADSD 8-21) | | 18121/175 | 1.15 (0.98-1.36) | 1.08 (0.91-1.28) | 1.05 (0.83-1.33) |  |
| 1. **ACR and HADS-A, n=9317** | |  |  |  |  |  |
| ACR no (ACR <3), n=8773 | | 127971/959 | Reference | Reference | Reference |  |
| ACR yes (ACR 3-30), n=1237 | | 12997/203 | 1.57 (1.34-1.85) | 1.38 (1.17-1.63) | 1.50 (1.18-1.90) |  |
| Anxiety Symptoms, no (HADS-A<8), n=8673 | | 120738/943 | Reference | Reference | Reference |  |
| Anxiety Symptoms, yes (HADS-A 8-21) | | 22844/173 | 1.17 (0.98-1.38) | 1.08 (0.91-1.29) | 1.10 (0.87-1.39) |  |
| 1. **ACR and highest score on HADS-D or HADS-A, n=9570** | |  |  |  |  |  |
| ACR, no (ACR <3), n=8773 | | 127971/959 | Reference | Reference | Reference |  |
| ACR, yes (ACR 3-30), n=1237 | | 12997/203 | 1.60 (1.36-1.87) | 1.39 (1.17-1.64) | 1.47 (1.16-1.86) |  |
| Anxiety or depression symptoms, no, n=8258 | | 114481898 | Reference | Reference | Reference |  |
| Anxiety or depression symptoms, yes, n=2282 | | 31554/259 | 1.10 (0.95-1.26) | 1.02 (0.88-1.18) | 0.99 (0.81-1.22) |  |
|  | Model 1: Age as underlying time scale and Sex as strata | | | | |  |
|  | Model 2: Model 1 + The Framingham variables diabetes (yes/no), Antihypertensive treatment (yes/no), Systolic Blood Pressure (mmHg), Smoking Status (never, previous, current), , total cholesterol (mmol/L), HDL cholesterol (mmol/L) | | | | |  |
|  | Model 3: Model2+ Estimated glomerulus filtration rate (mL/min), Body Mass Index (weight/m^2^), Waist Circumference (cm) , , Hard Exercise (none, <1 hr/week, 1-2 hr/week, >3 hr/week), Education or work (<=10 years school or unskilled worker , 10-12 years school or intermediate working class, >12 years of school or salariat working class) | | | | |  |

| Supplementary Table 2. Mechanisms related to missing on the physical activity question | | |
| --- | --- | --- |
| Variable | Without missing on physical activity, n=665 | With missing on physical activity, n=4355 |
| From random 5% selection, n (%) | 2972 (44.6) | 1386 (31.8) |
| Age mean (SD) | 54.3 (16.0) | 62.6 (13.8) |
| Sex, Men n (%) | 3185 (47.8) | 1595 (36.6) |
| Systolic Blood Pressure (mmHg) | 143.8 (23.0) | 150.8 (24.3) |
| Diabetes, n (%) | 838 (12.6) | 715 (16.4) |
| Blood Pressure Medication, n (%) | 3194 (48.0) | 2616 (60.1) |
| Education HUNT2 (years) or work (class) HUNT3, n (%) |  |  |
| 10 years of school or less or unskilled worker | 2688 (41.9) | 2211 (58.8) |
| 10-12 years of school or intermediate working class | 2666 (41.6) | 1226 (32.6) |
| >12 years of School or salariat working class | 1055 (16.5) | 322 (8.8) |
| Estimated glomerulus filtration rate, ml/min (mean, SD) | 92.4 (19.5) | 84.6 (18.4) |
| Albumine Creatinine Ratio 3-30, n (%) | 633 (10.5) | 604 (15.3) |
| HADS Depression >8, n % | 810 (12.5) | 539 (13.5) |
| HADS-Anxiety >8, n% | 1009 (15.7) | 591 (15.4) |
| Acute Myocardial Infarction, n (%) | 656 (9.8) | 578 (13.3) |
|  |  |  |

| **Supplementary Table 3.** Number (n, %) of participants* without and with Acute Myocardial Infarction (AMI) in Complete Case | | | |
| --- | --- | --- | --- |
|  | Event free | AMI | p |
| **ACR and Depression, n=9544** |  |  | <0.000 |
| ACR<3 and HADS-D<8 | 6561 (89.6) | 760 (10.3) |  |
| ACR<3 and HADS-D>=8 | 943 (87.5) | 135 (12.5) |  |
| ACR>=3 and HADS-D<8 | 839 (84.5) | 154 (15.5) |  |
| ACR>=3 and HADS-D>=8 | 120 (78.9) | 32 (21.1) |  |
| **ACR and Anxiety, n=9317** |  |  | <0.000 |
| ACR<3 and HADS-A<8 | 6187 (89.4) | 734 (10.6) |  |
| ACR<3 and HADS-A>=8 | 1143 (89.3) | 137 (10.7) |  |
| ACR>=3 and HADS-A<8 | 804 (84.3) | 150 (15.7) |  |
| ACR>=3 and HADS-A>=8 | 134 (82.7) | 28 (17.3) |  |
| **ACR and Anxiety or Depression, n=9575** |  |  | <0.000 |
| ACR<3 and HADS-D or HADS-A<8 | 5891 (89.4) | 701 (10.6) |  |
| ACR<3 and HADS-D or HADS-A>=8 | 1632 (89.0) | 202 (11.0) |  |
| ACR>=3 and HADS-D or HADS-A<8 | 768 (84.3) | 143 (15.7) |  |
| ACR>=3 and HADS-D or HADS-A>=8 | 195 (81.9) | 1. 18.1) |  |
| *N in each category of exposures. Abbreviations ACR=Albumin Creatinine Ratio measured in urine (mg/mmol). Anxiety and Depression Symptoms measured by the Hospital Anxiety and Depression Scale. | | | |
